# Structure-Based Design of Potent Peptidomimetic Inhibitors Covalently Targeting SARS-CoV-2 Papain-like Protease

**DOI:** 10.3390/ijms24108633

**Published:** 2023-05-11

**Authors:** Qian Wang, Guofeng Chen, Jian He, Jiameng Li, Muya Xiong, Haixia Su, Minjun Li, Hangchen Hu, Yechun Xu

**Affiliations:** 1 School of Chinese Materia Medica, Nanjing University of Chinese Medicine, Nanjing 210023, China; 2 State Key Laboratory of Drug Research, Shanghai Institute of Materia Medica, Chinese Academy of Sciences, Shanghai 201203, China; 3 University of Chinese Academy of Sciences, Beijing 100049, China; 4 Shanghai Synchrotron Radiation Facility, Shanghai Advanced Research Institute, Chinese Academy of Sciences, Shanghai 201210, China; 5 School of Pharmaceutical Science and Technology, Hangzhou Institute for Advanced Study, University of Chinese Academy of Sciences, Hangzhou 310024, China

**Keywords:** SARS-CoV-2, papain-like protease, covalent inhibitor, co-crystal structure, in vitro assay

## Abstract

The papain-like protease (PL^pro^) of severe acute respiratory syndrome coronavirus 2 (SARS-CoV-2) plays a critical role in the proteolytic processing of viral polyproteins and the dysregulation of the host immune response, providing a promising therapeutic target. Here, we report the structure-guide design of novel peptidomimetic inhibitors covalently targeting SARS-CoV-2 PL^pro^. The resulting inhibitors demonstrate submicromolar potency in the enzymatic assay (IC_50_ = 0.23 μM) and significant inhibition of SARS-CoV-2 PL^pro^ in the HEK293T cells using a cell-based protease assay (EC_50_ = 3.61 μM). Moreover, an X-ray crystal structure of SARS-CoV-2 PL^pro^ in complex with compound **2** confirms the covalent binding of the inhibitor to the catalytic residue cysteine 111 (C111) and emphasizes the importance of interactions with tyrosine 268 (Y268). Together, our findings reveal a new scaffold of SARS-CoV-2 PL^pro^ inhibitors and provide an attractive starting point for further optimization.

## 1. Introduction

The ongoing pandemic of COVID-19 (Coronavirus Disease-2019) has inflicted an unprecedented number of infections and fatalities worldwide [1]. This highly contagious disease is caused by a novel severe acute respiratory syndrome coronavirus 2 (SARS-CoV-2) which could result in severe respiratory diseases as well as unclear sequelae [2]. Although current vaccines and treatments have demonstrated some effectiveness in preventing COVID-19 or reducing its severity [3,4], a multitude of noteworthy Omicron variants has still emerged in many countries [5,6,7], necessitating the continued development of therapeutic strategies and drug candidates to combat SARS-CoV-2 [8].

The optimal antiviral drugs prefer to target the viral proteins essential for the SARS-CoV-2 life cycle. The papain-like protease (PL^pro^) and chymotrypsin-like protease (called 3CL^pro^, also referred to as main protease) are two essential cysteine proteases encoded by the SARS-CoV-2 genome. Both of them are required to cleave two viral polyproteins (pp1a and pp1ab) to generate 16 mature nonstructural proteins for genome transcription and replication [9,10]. In addition, SARS-CoV-2 PL^pro^ recognizes and cleaves the C-terminal LxGG sequence of ubiquitin (Ub) and ubiquitin-like proteins (UbLs) such as interferon-stimulated gene product 15 (ISG15) to remove the Ub and UbL modifications from host proteins, respectively [11,12]. The deubiquitinase (DUB) activity of SARS-CoV-2 PL^pro^ is thought to induce dysregulation in the host immune response against viral infection and replication [13,14,15]. Therefore, targeting SARS-CoV-2 PL^pro^ is an attractive strategy for both suppressing the virus replication and preventing the disruption of the host antiviral immunity [16].

Over the past decade, rational covalent drug design has attracted extensive attention from pharmaceutical chemists and led to the discovery of numerous approved covalent drugs [17,18]. Both SARS-CoV-2 3CL^pro^ and PL^pro^ have a catalytic cysteine that can be attacked by electrophiles, serving as ideal targets for covalent inhibitor design [19]. At present, two covalent inhibitors of SARS-CoV-2 3CL^pro^, nirmatrelvir and simnotrelvir, have been approved for the treatment of COVID-19 [3]. In contrast, there are few covalent inhibitors of SARS-CoV-2 PL^pro^ reported, which only include two peptidomimetic inhibitors (VIR250 and VIR251) [20] and GRL0617 analogs [21]. Although the GRL0617 analogs were much more potent relative to peptidomimetic inhibitors, their crystal structures in complex with SARS-CoV-2 PL^pro^ are not available yet. Accordingly, the peptidomimetic inhibitors were selected as the starting point for further optimization with the objective of identifying novel covalent inhibitors targeting SARS-CoV-2 PL^pro^.

In this work, we have identified a new class of covalent peptidomimetic inhibitors of SARS-CoV-2 PL^pro^ using a structure-guide design strategy. The most potent inhibitor displays a submicromolar potency in an enzymatic assay. Moreover, a crystal structure of SARS-CoV-2 PL^pro^ in complex with compound **2** was determined to understand the explicit binding mode of these congeneric inhibitors. In addition, the cell-based inhibitory activity of all designed compounds against SARS-CoV-2 PL^pro^ was tested by the PL-FlipGFP assay, a cell-based protease inhibition assay. Overall, our study comprehensively explored the in vitro inhibitory activities of novel covalent peptidomimetic inhibitors of SARS-CoV-2 PL^pro^ through biochemical and biophysical approaches.

## 2. Results

### 2.1. A Fusion of VIR251 and Compound ***1*** Led to the Design of Compound ***2***

**Figure 1:**
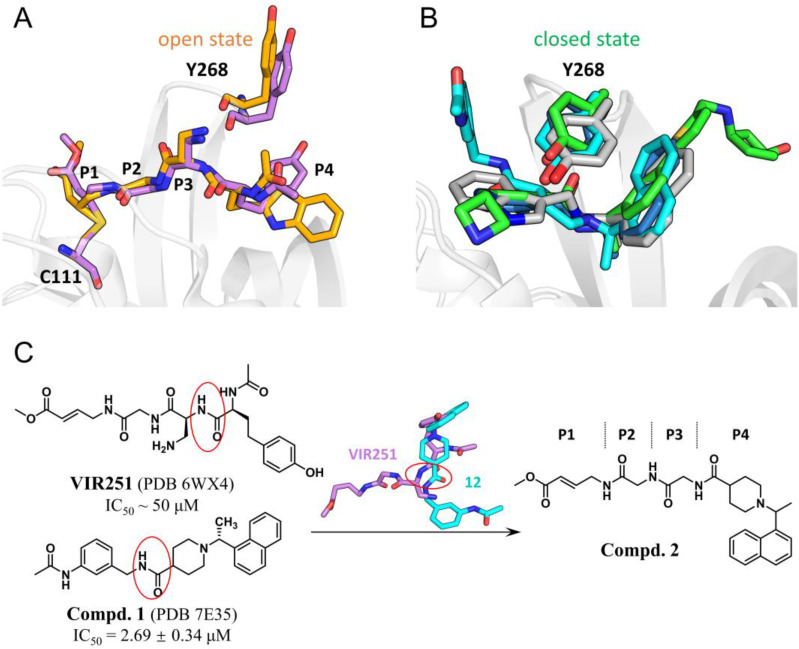
Design of compound **2** by a fusion of VIR251 and compound **1**. (**A**) Crystal structures of SARS-CoV-2 PL^pro^ bound with VIR250 (orange) and VIR251 (purple) showing an open conformation for the side chain of Y268. (**B**) Crystal structures of SARS-CoV-2 PL^pro^ bound with compound **1** (cyan), XR8-84 (green), GRL0617 (blue), and Jun9-84-3 (gray) showed a closed side-chain conformation of Y268. (**C**) Schematic description for the design of compound **2** based on VIR251 and compound **1**. The red circles highlight the amide bonds of VIR251 and compound **1**, and this amide bond of VIR251 is proximal to the one of compound **1**.

VIR250 and VIR251, as the first reported peptidomimetic inhibitors, covalently modify the catalytic cysteine (C111) and occupy the substrate binding pocket of SARS-CoV-2 PL^pro^ (Figure 1A). However, these two compounds show micromolar potency with a half-maximal inhibitory concentration (IC_50_) value of ~50 μM [20]. After a thorough investigation of the crystal structures of SARS-CoV-2 PL^pro^ in complex with VIR250 and VIR251, we found an open conformation for the side chain of tyrosine 268 (Y268) which has no interactions with the aromatic ring at the P4 position of these peptidomimetic inhibitors. In contrast, a number of crystal structures of SARS-CoV-2 PL^pro^ in complex with potent noncovalent inhibitors reveal a closed side-chain conformation of Y268 which forms distinct π-π interactions with the inhibitors in an edge-to-face manner (Figure 1B) [22,23,24,25]. Therefore, it’s reasonable to speculate that the replacement of the P4 group of VIR251 with the portion of noncovalent inhibitors could achieve more interactions with Y268 in the closed side-chain conformation and subsequently improve the potency of peptidomimetic inhibitors.

To test this prediction, we first overlaid the crystal structures of SARS-CoV-2 PL^pro^ in complex with VIR251 as well as previously reported noncovalent inhibitors to dig out a more suitable scaffold. To our delight, we found that compound **1** could be a good starting point [21], as its amide bond is proximal to the amide bond between the P3 and P4 groups of VIR251 (red circles shown in Figure 1C). As a result, compound **2** is designed and synthesized based on the fusion of VIR251 and compound **1**.

### 2.2. Compound ***2*** Showed Strong Inhibition against SARS-CoV-2 PL^pro^ In Vitro

**Figure 2:**
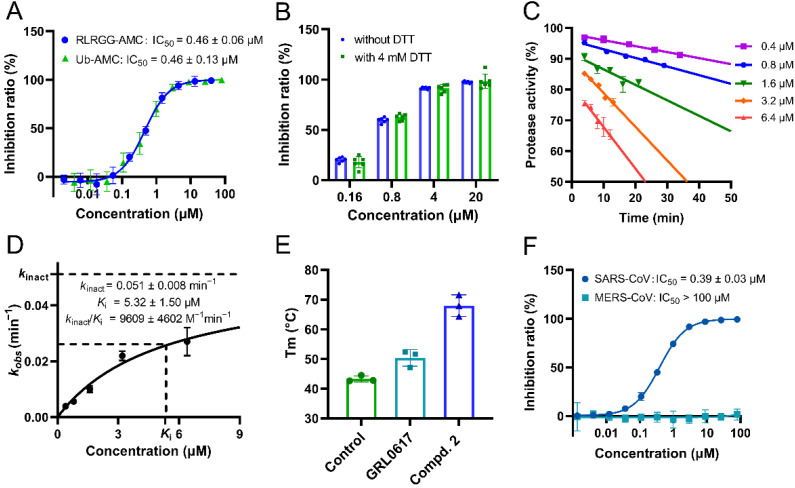
Characterization of compound **2** binding to SARS-CoV-2 PL^pro^. (**A**) The inhibition profiles for compound **2** against SARS-CoV-2 PL^pro^ using RLRGG-AMC and Ub-AMC substrates. (**B**) The inhibition of compound **2** against SARS-CoV-2 PL^pro^ in the presence or absence of 4 mM DTT. (**C**,**D**) The time-dependent inhibition of SARS-CoV-2 PL^pro^ by compound **2** at various concentrations, and the calculated covalent binding kinetic parameters. (**E**) *T*_m_ values of SARS-CoV-2 PL^pro^ incubated with DMSO (control) or different compounds in thermal shift assay. (**F**) The inhibition profiles for compound **2** against SARS-CoV and MERS-CoV PL^pro^ using the RLRGG-AMC substrate. Error bars represent mean ± error of three independent experiments in (**A**–**F**).

A short fluorogenic peptide substrate (RLRGG-AMC) was used to measure the inhibitory potency of compound **2** against SARS-CoV-2 PL^pro^ by an enzymatic assay [24,26]. As anticipated, compound **2** significantly inhibited the protease’s activity in a dose-dependent manner. The resulting IC_50_ value of compound **2** is 0.46 μM (Figure 2A), which is about 100-fold and 5-fold improvement in potency compared to that of VIR251 and compound **1**, respectively. We also performed the same assay to determine the IC_50_ value (1.47 μM) of GRL0617, the positive control (Figure S1), which is consistent with the one published [27,28]. Accordingly, the potency of compound **2** even exhibits a 2-fold increase compared to the positive control. It has been suggested in previous literature that reducing reagents such as dithiothreitol (DTT) in the enzymatic assay buffer can rule out promiscuous compounds that have non-specific inhibition towards many cysteine proteases [29,30]. For this reason, we carried out the enzymatic inhibition assay again in a DTT-containing buffer. As expected, there is no apparent change in the inhibitory activities of compound **2** at varied concentrations in the presence and absence of 4 mM DTT (Figure 2B), indicating its high specificity for SARS-CoV-2 PL^pro^. Additionally, considering the DUB activity of SARS-CoV-2 PL^pro^, an enzymatic inhibition assay was conducted using a second fluorogenic substrate (Ub-AMC) derived from Ub, which was synthesized according to the literature [31]. The result shows that compound **2** repeats the dose-dependent inhibition against the protease with an IC_50_ value of 0.46 μM (Figure 2A) which is identical to the one resulting from the enzymatic assay using RLRGG-AMC. These results together suggest that compound **2** is a potent peptidomimetic inhibitor of SARS-CoV-2 PL^pro^ while the fusion of VIR251 and compound **1** results in a strong synergetic effect on the inhibitory potency improvement.

The time-dependent inhibition assay was also performed to validate the covalent binding of compound **2**. Specifically, different concentrations of compound **2** were incubated with protease at the final concentration of 50 nM for the indicated time, and the activity of the protease in each reaction was measured after incubation. It turns out that compound **2** shows time-dependent inhibition of SARS-CoV-2 PL^pro^ (Figure 2C), which was in line with the model of covalent binding. Covalent ligand binding often involves a two-step process, an initial reversible binding event followed by the formation of a covalent bond, which is characterized by the binding affinity (*K*_i_) and the rate constant of covalent bond formation (*k*_inact_), respectively [32]. The resulting *K*_i_ and *k*_inact_ values of compound **2** are 5.32 μM and 0.051 min^−1^, respectively, and the ratio of these two parameters (*k*_inact_/*K*_i_) is 9609 M^−1^ min^−1^ (Figure 2D). Taken together, compound **2** is characterized as an effective peptidomimetic inhibitor targeting SARS-CoV-2 PL^pro^ in a covalent manner.

To confirm the specific binding between inhibitors and SARS-CoV-2 PL^pro^, a thermal shift assay (TSA) was also applied after incubation of 40 μM protease with 200 μM compounds. As shown in Figure 2E, the melting temperature (*T*_m_) of SARS-CoV-2 PL^pro^ incubated with compound **2** significantly increases compared to that of apo protease (68.0 °C vs. 43.3 °C) and it is also much higher than that of the protease incubated with GRL0617 (68.0 °C vs. 50.4 °C). These results demonstrate that compound **2** directly binds to SARS-CoV-2 PL^pro^ to greatly improve protein stability.

In addition, we also tested the inhibitory activity of compound **2** against the PL^pro^s from two other highly pathogenic human coronaviruses, SARS-CoV and middle east respiratory syndrome coronavirus (MERS-CoV). Compound **2** inhibits SARS-CoV PL^pro^ with an IC_50_ value of 0.39 μM (Figure 2F) which is similar to its potency against SARS-CoV-2 PL^pro^ (IC_50_ = 0.47 μM). However, compound **2** is basically ineffective towards MERS-CoV PL^pro^ even at a concentration of 100 μM (Figure 2F). The reason for such difference may be that SARS-CoV-2 PL^pro^ shares a sequence identity of 82.9% with SARS-CoV PL^pro^ while only 32.9% sequence identity with MERS-CoV PL^pro^ [16]. In this regard, compound **2** serves as a novel potent peptidomimetic inhibitor targeting both SARS-CoV and SARS-CoV-2 PL^pro^.

### 2.3. A Crystal Structure of SARS-CoV-2 PL^pro^ Covalently Bound with Compound ***2***

**Figure 3:**
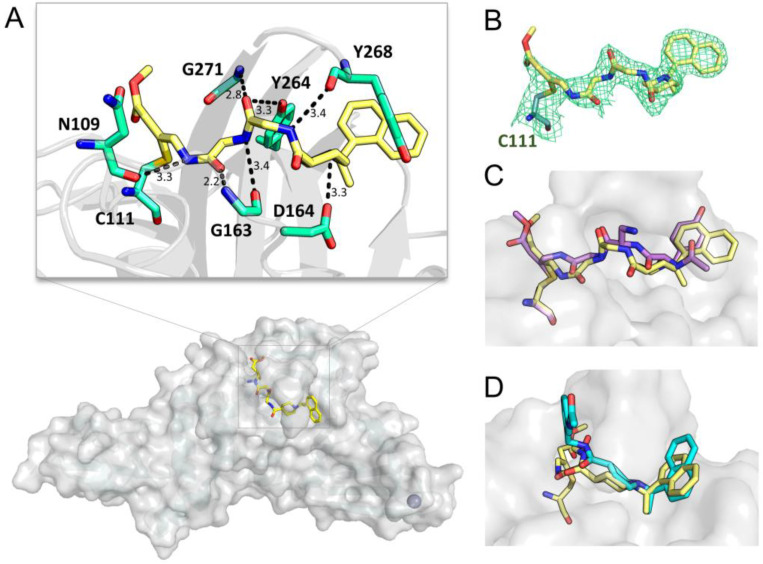
A crystal structure of SARS-CoV-2 PL^pro^ in complex with compound **2**. (**A**) Overall structure and interactions between the active-site residues (green) and compound **2** (yellow). The distance is labeled (Å). (**B**) The 2*F*o–*F*c electron density map of compound **2** contoured at 1.2 σ. (**C**,**D**) Crystal structure superimposition of compound **2** (yellow) on VIR251 (purple) or compound **1** (cyan) in complex with SARS-CoV-2 PL^pro^.

To gain insights into the precise ligand binding mode, a crystal structure of SARS-CoV-2 PL^pro^ in complex with compound **2** was determined at 2.5 Å resolution (Figure 3A, Table 1). As expected, the contiguous electron density is clearly shown between C111 and the P1 group of compound **2** (Figure 3B), demonstrating the covalent bond formed between the protease and **2**. In addition, the P1–P3 groups of compound **2** form multiple hydrogen bonds (H-bonds) with the main chain of N109, G163, Y268, and G271 as well as the side chain of Y264 (Figure 3A). The superimposed crystal structures of SARS-CoV-2 PL^pro^ in complex with compound **2** and VIR251 reveal that the protein-ligand interaction patterns of the P1–P3 groups of two inhibitors are highly conserved (Figure 3C). Meanwhile, the side chain of Y268 is in a closed conformation as anticipated so as to form π-π interactions with the P4-naphthyl group of compound **2**; a salt bridge is also established between the side chain of D164 and the piperidine ring of the P4-position (Figure 3A). The superimposition of crystal structures of SARS-CoV-2 PL^pro^ bound with compounds **1** and **2** reveals that the P4 group of the two compounds employs a highly similar binding pose in the complexes (Figure 3D). Therefore, in the SARS-CoV-2 PL^pro^-**2** complex, two fragments individually adopted from VIR251 and compound **1**, bind to the pocket as we desired, highlighting the elegance and utility of the structure-based drug design in significantly facilitating the development of new inhibitors.

### 2.4. Substitution of the Warhead Leading to the Design of Compound ***4***

**Figure 4:**
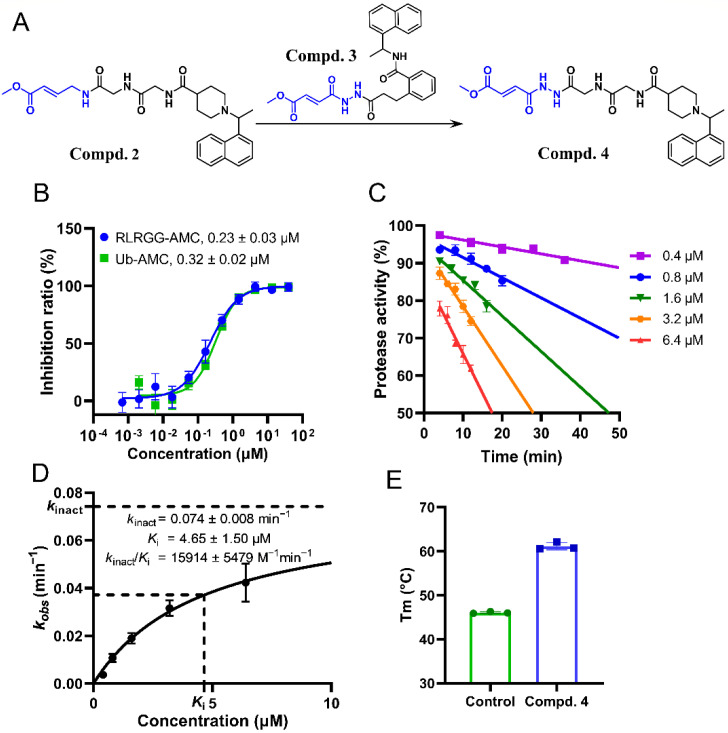
Characterization of compound **4** binding to SARS-CoV-2 PL^pro^. (**A**) Schematic description for the design of compound **4** based on compounds **2** and **3**. (**B**) The inhibition profiles for compound **4** against SARS-CoV-2 PL^pro^ using RLRGG-AMC and Ub-AMC substrates. (**C**,**D**) Time-dependent inhibition of SARS-CoV-2 PL^pro^ by compound **4** at various concentrations, and the calculated covalent binding kinetic parameters. (**E**) *T*_m_ values of SARS-CoV-2 PL^pro^ incubated with DMSO (control) and compound **4** determined by the thermal shift assay. Error bars represent mean ± error of three independent experiments in (**B**–**E**).

To further improve the potency of compound **2**, we borrowed the warhead from compound **3** as it is a GRL0617 analog and possesses strong inhibition against SARS-CoV-2 PL^pro^ (IC_50_ = 94 nM) [21]. As a result, compound **4** was designed and synthesized (Figure 4A). The enzymatic inhibition assay displays that the inhibitory potency of compound **4** is ~1-fold higher than **2**. The exact IC_50_ values measured by using RLRGG-AMC and Ub-AMC substrates are 0.23 μM and 0.32 μM, respectively (Figure 4B). In addition, compound **4** inhibits SARS-CoV-2 PL^pro^ in a time-dependent manner (Figure 4C), suggesting that a covalent binding mode is utilized by compound **4**. Kinetic parameters of compound **4** binding with SARS-CoV-2 PL^pro^ were also calculated and the resulting value of *k*_inact_/*K*_i_ is 15,914 M^−1^ min^−1^ which also showed an increase compared to compound **2** (Figure 4D). This improvement is mainly attributed to the substitution of the warhead as the *k*_inact_ value of compound **4** is higher than that of compound **2** (0.074 min^−1^ vs. 0.051 min^−1^) while the *K*_i_ values of the two compounds are similar (4.65 μM vs. 5.32 μM, Figure 2D and Figure 4D). Moreover, the *T*_m_ value of SARS-CoV-2 PL^pro^ incubated with compound **4** has a 15.1 °C increase compared to that of apo protease, which confirms the direct binding between the compound and the protease (Figure 4E). Consequently, compound **4** is a new covalent peptidomimetic inhibitor of SARS-CoV-2 PL^pro^ with improved potency compared to compound **2**.

### 2.5. The Cell-Based Protease and Cytotoxicity Assay of Two Designed Compounds

**Figure 5:**
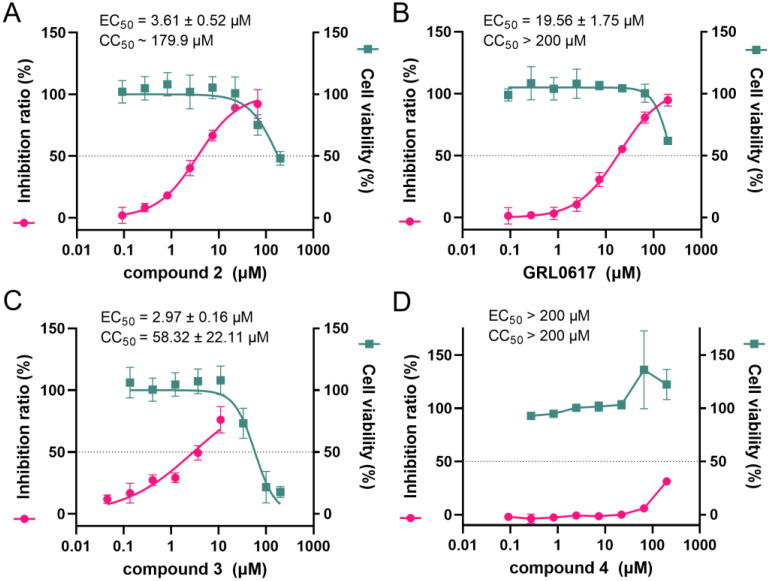
Intracellular inhibition and cytotoxicity of compounds **2**–**4** and GRL0617. Dose-response curves of compound **2** (**A**), GRL0617 (**B**), compound **3** (**C**) and compound **4** (**D**) in the PL-FlipGFP assays (rose) and the CCK8 assays (green) using HEK293T cells. Error bars represent mean ± error of three independent experiments.

The intracellular inhibition of two designed compounds was tested using the PL-FlipGFP assay, a cell-based protease assay initially developed by Wang et al. [25]. In this assay, a fluorogenic GFP containing a cleavage site (LRGGAPTK) for SARS-CoV-2 PL^pro^, namely PL-FlipGFP, and a red fluorescent protein (mCherry) were constructed together. The ratio of GFP/mCherry fluorescent signal was used to represent the activity of the protease in cells and reveal the intracellular inhibition of compounds. The results show that compound **2** displays potent intracellular inhibition with the half-maximal effective concentration (EC_50_) of 3.61 μM (Figure 5A), which exhibits ~4.4-fold improvement compared to GRL0617 (EC_50_ = 19.56 μM, Figure 5B) and comparable potency relative to compound **3** (EC_50_ = 2.97 μM, Figure 5C) that is the so far most potent covalent inhibitor of SARS-CoV-2 PL^pro^. Unfortunately, compound **4** shows remarkable inhibition against the protease in the enzymatic assay, but it hardly has an inhibitory effect in this cell-based protease assay (Figure 5D). In addition, we tested the cytotoxicity of these mentioned inhibitors in HEK293T cells by the cell counting kit-8 (CCK8) assay. The results show that the half-maximal cytotoxic concentration (CC_50_) value of compound **2** is ~180 μM (Figure 5A). Collectively, compound **2** is a promising covalent peptidomimetic inhibitor of SARS-CoV-2 PL^pro^ with potent intracellular inhibition and low cytotoxicity.

## 3. Discussion

The persistent pandemic COVID-19 together with previous epidemics of SARS and MERS has raised great awareness of the increasing infection risks of highly pathogenic CoVs. This concern calls for a huge demand for the discovery and development of anti-CoV drugs. In this work, using a fusion strategy guided by the structural information, we identified the novel peptidomimetic inhibitors covalently targeting SARS-CoV-2 PL^pro^, which is also well verified by the determined crystal structure. Meanwhile, the inhibitors such as compound **2** exhibit a submicromolar potency (IC_50_ = 0.46 μM) in the enzymatic assay and a significant inhibition (EC_50_ = 3.61 μM) in the cell-based protease assay (Figure S2).

The best inhibitor reported in the previous publication was also tested in our study [21], and it is referred to as compound **3**. We found that the potency of our inhibitors is comparable to compound **3** (IC_50_ = 0.094 μM, EC_50_ = 2.97 μM). In addition, our compound has lower cytotoxicity in HEK293T cells (CC_50_ = 179.9 μM vs. 58.3 μM), indicating a higher safety profile of the compound. Therefore, a class of novel and potent inhibitors against SARS-CoV-2 PL^pro^ has been identified in our study, which is valuable for further development.

It is noteworthy that our work presented here used a structure-based drug design approach to successfully design a cysteine-targeted covalent ligand. The structure information reveals the importance of Y268 side-chain conformation in protein-ligand interactions and allows us to efficiently obtain a potent covalent peptidomimetic inhibitor using a fusion strategy. The resulting compound **2** is more potent than both parental compounds, which indicates the strong synergetic effect of two fragments individually from two parental compounds to improve the potency of the new compound is achieved. To gain further insight into the protein-ligand interactions involving Y268 and other surrounding residues, computational studies are needed for the optimization of the inhibitors. Accordingly, the present study highlights the power and advantage of the structure-based drug design strategy.

In summary, we report a class of novel and potent peptidomimetic inhibitors that covalently target SARS-CoV-2 PL^pro^. Our findings present a new scaffold of SARS-CoV-2 PL^pro^ inhibitors and provide a promising starting point for further optimization.

## 4. Materials and Methods

### 4.1. Materials and Methods for Synthesis and Characterization of Compound ***2*** and ***4***

All chemical reagents and compounds were used as supplied by standard suppliers (Bidepharm, Shanghai, China) without further purification. All reactions were monitored by thin layer chromatography (TLC), and visualization was achieved by using ultraviolet light (254 nm). The column chromatography was performed using flash chromatography. ^1^H NMR and ^13^C NMR spectra were recorded on a BRUKER AVANCE NEO 500 or 400 (Billerica, MA, USA) at 500 or 400 MHz and 126 or 100 MHz, respectively. Coupling constants (*J*) are expressed in hertz. Chemical shifts (*δ*) of NMR spectra are reported in parts per million (ppm) units. Mass spectra (MS) of compounds were measured using a Thermo Fisher FINNIGAN LTQ spectrometer (Thermo Finnigan, San Jose, CA, USA). The purity of all tested compounds was confirmed to be ≥95% by HPLC. Analysis was performed on a EClassical 3100 HPLC system (Elite, Dalian, China) under the following analytical method: column, WondaSil Superb C18 (5 μm, 4.6 mm × 250 mm); solvent A: water containing 1‰ trifluoroacetic acid (TFA); solvent B: acetonitrile containing 1‰ TFA; gradient, 10% B to 100% B over 15 min, 100% B for 5 min; flow rate, 1 mL/min; detective wavelength, 220 nm, 254 nm; column temperature, 25 °C. The spectroscopic graphs are provided in the Supplementary Materials (Figures S3–S16).

#### Synthesis Procedure

*Methyl 1-(1-(Naphthalen-1-yl)ethyl)piperidine-4-carboxylate (***2b***).* Under N_2_ atmosphere and in an ice bath 1-(naphthalen-1-yl)ethan-1-ol (**2a**) (1.00 g, 5.80 mmol) was dissolved in the mixture of dichloromethane (15 mL), DIPEA (2.88 mL, 17.42 mmol) and molecular sieves (4 Å, 8–12 mesh). Then a solution of Ms_2_O (1.31 g, 7.55 mmol) dissolved in dichloromethane (2 mL) was added into the mixture dropwise and the mixture was stirred in the ice bath for 40 min. After that, methyl piperidine-4-carboxylate (4.71 mL, 34.84 mmol) was added to the mixture dropwise under ice bath, then the brown mixture was stirred at r.t. for 16 h. Molecular sieves were removed by filtration, and the filtrate was extracted with water (20 mL) and dichloromethane (3 × 20 mL). Then combined organic layers were washed with saturated NH_4_Cl solution and brine, dried over anhydrous Na_2_SO_4_ and filtered. The solvent was removed under reduced pressure, and the residue was purified by a flash column chromatography on silica (99% petroleum ether (PE) in ethyl acetate (EtOAc) to 25% PE in EtOAc) to yield **2b** as a pale yellow oily substance (1.1 g, 63.95% yield). HRMS-ESI (*m/z*): 298.1796 [M + H]^+^ for C_19_H_24_NO_2_; ^1^H NMR (500 MHz, Chloroform-*d*) *δ* 8.46–8.40 (m, 1H), 7.85–7.81 (m, 1H), 7.74–7.70 (m, 1H), 7.56 (dd, *J* = 7.2, 1.3 Hz, 1H), 7.49–7.43 (m, 2H), 7.41 (dd, *J* = 8.2, 7.2 Hz, 1H), 4.08 (q, *J* = 6.7 Hz, 1H), 3.64 (s, 3H), 3.16–3.07 (m, 1H), 2.84–2.78 (m, 1H), 2.31–2.24 (m, 1H), 2.11–2.02 (m, 2H), 1.93–1.88 (m, 1H), 1.79–1.73 (m, 2H), 1.72–1.65 (m, 1H), 1.45 (d, *J* = 6.7 Hz, 3H). ^13^C NMR (126 MHz, CDCl_3_) *δ* 175.86, 140.79, 134.04, 131.66, 128.66, 127.26, 125.42, 125.37, 125.29, 124.49, 124.28, 72.14, 61.63, 51.53, 51.51, 49.13, 41.37, 28.70, 28.66, 18.62.

*1-(1-(Naphthalen-1-yl)ethyl)piperidine-4-carboxylic acid (***2c***)*. In a solution of methyl 1-(1-(naphthalen-1-yl)ethyl)piperidine-4-carboxylate (**2b**) (600 mg, 2.02 mmol) dissolved in methanol (6 mL), a solution of NaOH (323 mg, 8.07 mmol) dissolved in H_2_O (3 mL) was added, and the mixture was stirred at 70 °C for 1 h. The solution was concentrated and acidified to pH 2~3 and poured into the violently stirred ice water (20 mL). After 30 min, the suspension was filtrated and the precipitate was wash with ice water to yield **2c** as a white solid (320 mg, 55.97% yield), which was dried and directly used in the next step without further purification.

*Methyl (1-(1-(Naphthalen-1-yl)ethyl)piperidine-4-carbonyl)glycylglycinate (***2d***)*. 1-(1-(Naphthalen-1-yl)ethyl)piperidine-4-carboxylic acid (**2c**) (100 mg, 0.336 mmol), methyl glycylglycinate hydrochloride (68 mg, 0.370 mmol), HATU (154 mg, 0.404 mmol) were added into dichloromethane (4 mL), then DIPEA (270 μL, 1.68 mmol) was added into the stirred mixture. The mixture was stirred at r.t. for 10 h. Then the solution was extracted with water (20 mL) and dichloromethane (3 × 20 mL). After the solvent was removed under reduced pressure, the crude material was subject to column chromatography (silica, DCM/MeOH = 1/20) to give **2d** as an off-white solid (137 mg, 95.80% yield). HRMS-ESI (*m/z*): 412.2235 [M + H]^+^ for C_23_H_30_N_3_O_4_; ^1^H NMR (500 MHz, Chloroform-*d*) *δ* 8.47–8.40 (m, 1H), 7.87–7.81 (m, 1H), 7.73 (d, *J* = 8.2 Hz, 1H), 7.56 (d, *J* = 7.1 Hz, 1H), 7.50–7.44 (m, 2H), 7.42 (t, *J* = 7.6 Hz, 1H), 6.70 (t, *J* = 5.5 Hz, 1H), 6.35 (t, *J* = 5.3 Hz, 1H), 4.10 (q, *J* = 6.7 Hz, 1H), 4.03 (d, *J* = 5.3 Hz, 2H), 3.97 (d, *J* = 5.2 Hz, 2H), 3.74 (s, 3H), 3.26–3.18 (m, 1H), 2.89 (dd, *J* = 11.6, 4.5 Hz, 1H), 2.21–2.12 (m, 1H), 2.10–1.99 (m, 2H), 1.86–1.72 (m, 4H), 1.46 (d, *J* = 6.6 Hz, 3H). ^13^C NMR (126 MHz, Chloroform-*d*) *δ* 175.98, 169.97, 169.31, 140.68, 134.07, 131.68, 128.69, 127.34, 125.50, 125.38, 125.34, 124.55, 124.29, 61.65, 52.44, 51.79, 49.10, 43.38, 43.03, 41.16, 29.22, 29.17, 18.59.

*(1-(1-(Naphthalen-1-yl)ethyl)piperidine-4-carbonyl)glycylglycine (***2e***)*. In a solution of methyl (1-(1-(naphthalen-1-yl)ethyl)piperidine-4-carbonyl)glycylglycinate (**2d**) (100 mg, 0.243 mmol) dissolved in methanol (3 mL), a solution of LiOH (23.5 mg, 0.972 mmol) dissolved in H_2_O (1 mL) were added, and the mixture was stirred at 70 °C for 1 h. The solution was concentrated, and the pH was adjusted to 2~3. Then, the solvent was removed under reduced pressure and the crude material was subject to column chromatography (silica, DCM/MeOH = 8/1, 1% HOAc) to give **2e** as a white oily substance (47 mg, 64.14% yield).

*N-(2-((2-Hydrazinyl-2-oxoethyl)amino)-2-oxoethyl)-1-(1-(naphthalen-1-yl)ethyl)piperdine-4-carboxamide (***4e***)*. In a solution of methyl (1-(1-(naphthalen-1-yl)ethyl)piperidine-4-carbonyl)glycylglycinate (**2d**) (45 mg, 0.109 mmol) dissolved in ethanol (3 mL), a solution of NH_2_-NH_2_·H_2_O (85%) (140 μL, 2.180 mmol) was added, and the mixture was stirred at 40 °C for 30 min. The resulting solid was removed by filtration and the solution was concentrated under reduced pressure to yield **4e**, which was used directly in the next step without further purification (24 mg, 53.33% yield).

*Methyl (E)-4-(2-(2-(1-(1-(Naphthalen-1-yl)ethyl)piperidine-4-carboxamido)acetamido)acetamido)but-2-enoate (***2***).* (1-(1-(Naphthalen-1-yl)ethyl)piperidine-4-carbonyl)glycylglycine (**2e**) (30 mg, 0.076 mmol), methyl (E)-4-aminobut-2-enoate hydrochloride (11.5 mg, 0.069 mmol), HATU (29.0 mg, 0.076 mmol) were added into N,N-dimethylformamide (2 mL), then DIPEA (114 μL, 0.685 mmol) was added into the stirred mixture. The mixture was stirred at r.t. for 10 h. Then the solution was extracted with water (20 mL) and ethyl acetate (3 × 20 mL). After the solvent was removed under reduced pressure, the crude material was subject to column chromatography (silica, DCM/MeOH = 1/8) to yield **2** as a pale yellow powder (23 mg, 68.66% yield). HRMS-ESI (*m/z*): 495.2608 [M + H]^+^ for C_27_H_35_N_4_O_5_; ^1^H NMR (500 MHz, Chloroform-*d*) *δ* 8.43–8.35 (m, 1H), 7.87–7.79 (m, 1H), 7.72 (d, *J* = 8.2 Hz, 1H), 7.54 (d, *J* = 7.1 Hz, 1H), 7.50–7.43 (m, 2H), 7.41 (t, *J* = 7.7 Hz, 1H), 7.34 (t, *J* = 6.2 Hz, 1H), 7.16 (t, *J* = 6.2 Hz, 1H), 6.89–6.75 (m, 2H), 5.88 (dd, *J* = 15.8, 2.0 Hz, 1H), 4.11 (q, *J* = 6.6 Hz, 1H), 3.98 (d, *J* = 5.7 Hz, 2H), 3.93 (d, *J* = 5.7 Hz, 2H), 3.88 (d, *J* = 5.3 Hz, 2H), 3.65 (s, 3H), 3.18 (d, *J* = 11.0 Hz, 1H), 2.89–2.81 (m, 1H), 2.18–2.11 (m, 1H), 2.08–1.98 (m, 2H), 1.83 (d, *J* = 12.8 Hz, 1H), 1.73–1.61 (m, 3H), 1.45 (d, *J* = 6.6 Hz, 3H). ^13^C NMR (126 MHz, Chloroform-*d*) *δ* 176.85, 170.14, 169.11, 166.64, 144.09, 140.46, 134.13, 131.74, 128.80, 127.47, 125.59, 125.45, 125.43, 124.61, 124.26, 121.38, 61.50, 51.73, 49.07, 43.54, 43.21, 43.13, 40.16, 29.15, 18.47.

*Methyl (E)-4-(2-((1-(1-(Naphthalen-1-yl)ethyl)piperidine-4-carbonyl)glycylglycyl)hydrazinyl)-4-oxobut-2-enoate (***4***).* N-(2-((2-Hydrazinyl-2-oxoethyl)amino)-2-oxoethyl)-1-(1-(naphthalen-1-yl)ethyl)piperidine-4-carboxamide (**4e**) (24 mg, 0.058 mmol), (E)-4-Methoxy-4-oxobut-2-enoic acid (9.1 mg, 0.070 mmol), HATU (33.2 mg, 0.087 mmol) were added into N,N-dimethylformamide (2 mL), then DIPEA (41 μL, 0.233 mmol) was added into the stirred mixture. The mixture was stirred at r.t. for 10 h, then the solution was extracted with water (20 mL) and ethyl acetate (3 × 20 mL). After the solvent was removed under reduced pressure, the crude material was subject to column chromatography (silica, DCM/MeOH = 1/10) to give **4,** an off-white powder (23 mg, 75.32% yield). HRMS-ESI (*m/z*): 524.2507 [M + H]^+^ for C_27_H_34_N_5_O_6_; ^1^H NMR (500 MHz, DMSO-*d*_6_) *δ* 10.56 (d, *J* = 2.3 Hz, 1H), 10.22 (d, *J* = 2.4 Hz, 1H), 9.49–9.38 (s, 1H), 8.40 (d, *J* = 8.6 Hz, 1H), 8.22–8.19 (m, 1H), 8.06–8.03 (m, 1H), 7.91 (d, *J* = 7.0 Hz, 1H), 7.73–7.58 (m, 3H), 7.05 (d, *J* = 15.6, 1H), 6.67 (d, *J* = 15.6 Hz, 1H), 5.44–5.38 (m, 1H), 3.96–3.92 (m, 1H), 3.80 (d, *J* = 5.9 Hz, 2H), 3.76–3.69 (m, 5H), 3.16–3.09 (m, 1H), 3.09–3.01 (m, 1H), 2.92–2.81 (m, 1H), 2.50–2.39 (m, 2H), 2.07–1.89 (m, 3H), 1.83–1.64 (m, 4H). ^13^C NMR (126 MHz, DMSO) *δ* 173.07, 169.07, 167.19, 165.10, 161.08, 134.77, 133.40, 132.30, 131.04, 129.67, 129.34, 128.96, 127.02, 126.22, 125.58, 122.86, 117.82, 59.24, 52.04, 49.71, 49.37, 41.66, 40.29, 38.62, 25.83, 25.74, 17.80.

### 4.2. Plasmids Construction

The cDNA encoding SARS-CoV-2 PL^pro^, SARS-CoV PL^pro^ and MERS-CoV PL^pro^ with a 6× His tag at N-terminus were cloned into a pET-15b-SUMO vector, respectively. Lb^pro^ (29–195) from foot-and-mouth disease virus with an 8× His tag at N-terminus was cloned into a pET-11d vector. The human Ub gene with a 6× His tag at C-terminus was cloned into a pET-28a vector. All cDNAs mentioned above were *E. coli.* codon-optimized and synthesized by GenScript (Shanghai, China).

For transfection of mammalian cells, the cDNA encoding SARS-CoV-2 PL^pro^ with mammalian codon optimization was also ordered from GenScript and cloned into the pcDNA3.1 with a C-terminal FLAG tag. The sequence of pcDNA3-PL-flipGFP-T2A-mCherry was designed based on the plasmid of pcDNA3-TEV-flipGFP-T2A-mCherry (Addgene catalog NO.124429) where TEV cleave site was replaced by SARS-CoV-2 PL^pro^ cleavage site (amino acid sequence: LRGGAPTK) and ordered from GenScript.

### 4.3. Expression and Purification of SARS-CoV, SARS-CoV-2 and MERS-CoV PL^pro^

The expression and purification processes of SARS-CoV-2 PL^pro^, SARS-CoV PL^pro^ and MERS-CoV PL^pro^ were similar as described below. Briefly, the expression plasmids were transformed into *E. coli.* Rosetta (DE3) competent cells. The cells were grown in LB medium to an OD_600_ of 0.8 and induced by IPTG at a final concentration of 0.5 mM and shaken at 18 °C overnight. The 6× His-SUMO2-SARS-CoV-2 PL^pro^ was first purified by the Ni-NTA column (GE Healthcare, Marlborough, MA, USA) and cleaved by SUMO Specific Peptidase 2. The resulting protein samples were further purified by SP-Sepharose (GE Healthcare) and Superdex75 (GE Healthcare). The eluted proteins were stored in a solution containing 25 mM HEPES (pH 7.5) and 2 mM DTT for the subsequent experiments.

### 4.4. Crystallization and Data Collection

SARS-CoV-2 PL^pro^ (1 mg/mL) was incubated with 100 μM peptidomimetic inhibitors in 25 mM HEPES (pH 7.5) at 10 °C for 2 h. The mixture was supplemented with 10 mM DTT and concentrated to 12 mg/mL for crystallization. The crystal of SARS-CoV-2 PL^pro^ in complex with compound **2** was grown by mixing equal volumes of the protein/compound and a reservoir (0.2 M sodium bromide, 20% *w*/*v* PEG 3350) on a 96-well sitting plate at 20 °C. Before data collection, crystals were flash-frozen in liquid nitrogen directly in the presence of the reservoir solution. X-ray diffraction data were collected at beamline BL19U1 at the Shanghai Synchrotron Radiation Facility and processed with the program autoPROC 1.0 [33]. The structure was solved by the program PHASER 2.8.3 [34] and refined with the program PHENIX 1.17.1-3660 [35]. The refined structure was deposited into the Protein Data Bank with accession code 8IHO. The complete statistics as well as the quality of the solved structures are given in Table 1.

### 4.5. Synthesis of the Fluorogeneic Substrate Ub-AMC Based on Ub

The expression process of Ub and Lb^pro^ was the same as that of SARS-CoV-2 PL^pro^. Both Ub and Lb^pro^ were first purified by a Ni-NTA column (GE Healthcare), followed by buffer exchange using a Superdex75 (GE Healthcare) pre-equilibrated with a buffer containing 50 mM HEPES, 150 mM NaCl, pH 8.0. The fractions were flash-frozen in liquid nitrogen and stored at −80 °C for further use. Ub-AMC was synthesized according to previous references [31]. Specifically, Gly-Gly-AMC was dissolved in DMSO to a saturated solution and mixed with an equal volume of Ub stock solution (5 mM). After that, Lb^pro^ (40 μM) was added to the reaction solution at room temperature. After incubating for about 6 h, the reaction mixture was purified by a Ni-NTA column (GE Healthcare). The flow-through was further purified by Superdex 75 (GE Healthcare) pre-equilibrated with a buffer containing 50 mM HEPES, 150 mM NaCl, and pH 8.0. The fraction of the second main peak in the chromatogram was collected and stored at −80 °C for further use.

### 4.6. Enzymatic Assays of SARS-CoV, SARS-CoV-2 and MERS-CoV PL^pro^

The enzymatic inhibition assays of all compounds were performed using 96-well plates at room temperature. The fluorogenic substrates used in the assay were RLRGG-AMC [36] which was synthesized by GenScript or Ub-AMC synthesized as described above. The assays were performed in a total volume of 120 μL, which contained the following components: 50 mM HEPES pH 7.0, 0.1 mg/mL BSA, 50 nM SARS-CoV-2 PL^pro^, indicated concentrations of the compound or an equal volume of solvent (DMSO or H_2_O). After 60 min incubation, reactions were initiated with the addition of RLRGG-AMC or Ub-AMC to reach a final concentration of 20 µM or 1.5 µM, respectively. After that, the fluorescent signal (excitation: 360 nm, emission: 460 nm) was immediately measured every 1 min for 5 min with a BioTek H1 plate reader (Winooski, VT, USA). The initial velocity of the reaction was obtained by fitting the linear portion of the curve into a straight line and the inhibition effect of compounds was evaluated by calculating the change of the initial rate. Half maximal inhibitory concentration (IC_50_) was determined by nonlinear regression analysis of the dose-response curves using GraphPad Prism 9.0. Three independent experiments were performed.

### 4.7. Kinetic Analysis of Compounds Covalently Binding to SARS-CoV-2 PL^pro^

The binding of covalent compounds with SARS-CoV-2 PL^pro^ can be described in two steps: an initial reversible binding event (*K*_i_) followed by the formation of the covalent bond (*k*_incat_). For determination of *K*_i_ and *k*_inact_, SARS-CoV-2 PL^pro^ at a final concentration of 50 nM was incubated with various concentrations of the compound for the indicated time. At each time point, the enzymatic assay was carried out as mentioned above. Relative enzymatic activity was calculated by the initial velocity ratio of reactions added with the compound over to the reaction added with solvent. Relative enzymatic activity for various concentrations of the compound over a time course was fitted to the semilogarithmic plot equation to generate observed the rate constant value (*k*_obs_) for each concentration. The resulting *k*_obs_ values were then plotted versus compound concentrations ([C]), then *k*_inact_ and *K*_i_ values were calculated according to the equation: *k*_obs_ = *k*_inact_ × ([C]/([C] + *K*_i_)) using GraphPad Prism 9.0. For each compound, three independent experiments were performed for the determination of *k*_inact_ and *K*_i_ values. Three independent experiments were performed.

### 4.8. Thermal Shift Assay of SARS-CoV-2 PL^pro^ Incubated with Compounds

Inhibitors binding to SARS-CoV-2 PL^pro^ was estimated by differential scanning fluorimetry via a Bioer Real-Time PCR QuantGene 9600 machine (Hangzhou, China). Specifically, SARS-CoV-2 PL^pro^ was diluted to a final concentration of 40 μM. Then, the diluted protein was added with 200 μM inhibitor and treated at 25 °C for 30 min. After that, 1 × SYPRO orange dye was added to the mixture, and the fluorescence was monitored under a temperature gradient range from 25 °C to 90 °C with 0.03 °C/s incremental step. The measured *T*_m_ was calculated from a melt curve with the program Gene-9660. Three independent experiments were performed.

### 4.9. Cell-Based PL-FlipGFP Assay

The cell-based PL-FlipGFP assay was designed stemming from previous reports [11,25]. Briefly, HEK293T cells were seeded in 6-well plates. The following day, cells at 60–80% confluency were co-transfected with 2 μg pcDNA3-PL-flipGFP-T2A-mCherry plasmid and 0.2 μg protease expression plasmid in each well with 6.6 μg polyethylenimine (PEI). Five hours later, the cells were digested and divided into 96-well plates to a confluence of 30–40%. Different concentrations of the compounds were added and then the cells were incubated at 37 °C for 48 h. On the third day, the supernatant was removed and 60 μL lysis buffer (100 mM Tris pH 7.5, 100 mM NaCl, 0.5% Triton X-100) was added. After 5 min of incubation at 4 °C, 40 μL cell lysate was transferred to a 384-well plate and the fluorescent signal of GFP (excitation: 475 nm, emission: 505 nm) and mCherry (excitation: 580 nm, emission: 610 nm) were measured. The inhibition ratio was calculated based on the ratio of GFP/mCherry fluorescent signal. The EC_50_ value was calculated by plotting the inhibition ratio over the applied compound concentration in GraphPad Prism 9.0. Three independent experiments were performed.

### 4.10. Cytotoxicity Assay

The cytotoxicity of compounds on the HEK293T cells was determined by cell counting kit-8 (CCK8) assays. HEK293T cells were treated with compounds at different concentrations. DMSO-treated cells were used as the control. After 24 h, the cytotoxicity was measured using CCK8 according to the manufacturer’s instructions (Yeasen, Shanghai, China). At final, the absorbance at a wavelength of 450 nm was measured using a BioTek H1 plate reader and the CC_50_ value was calculated in GraphPad Prism 9.0.

## Figures and Tables

**Table 1:** Crystallography data collection and refinement statistics.

	SARS-CoV-2 PL^pro^—2
PDB ID	8IHO
Wavelength	0.979
Resolution range	42.03–2.55 (2.641–2.55) *
Space group	*P 2_1_2_1_2_1_*
Cell Dimension (a, b, c)	118.629 Å, 137.35 Å, 59.571 Å
Total reflections	359,709 (37,533)
Unique reflections	32,494 (3191)
Multiplicity	11.1 (11.8)
Completeness (%)	99.84 (99.91)
Mean I/sigma (I)	12.90 (2.32)
R-merge	0.1449 (1.162)
CC1/2	0.997 (0.693)
Reflections used in refinement	32,478 (3191)
Reflections used for R-free	1579 (145)
R-work	0.2543 (0.3788)
R-free	0.3047 (0.4075)
Number of non-hydrogen atoms	4892
macromolecules	4818
ligands	74
RMS (bonds)	0.003
RMS (angles)	0.55
Ramachandran favored (%)	96.58
Ramachandran allowed (%)	3.42
Ramachandran outliers (%)	0.00
Clashscore	6.21
MolProbity Score	1.56
Average B-factor	56.41
macromolecules	56.48
ligands	51.88

* Statistics for the highest-resolution shell are shown in parentheses.

## Data Availability

Data are contained within the article and Supplementary Materials.

## Supplementary Materials

The supporting information can be downloaded at: https://www.mdpi.com/article/10.3390/ijms24108633/s1.
