# Supplementary material for: Structure-Based Design of Potent Peptidomimetic Inhibitors Covalently Targeting SARS-CoV-2 Papain-like Protease
Source: Int J Mol Sci. 2023 May 11;24(10):8633. doi: 10.3390/ijms24108633 (PMC10218254; doi:10.3390/ijms24108633)
Supplement: Supplementary file 1 [file ijms-24-08633-s001.zip › ijms-2279759-supplementary.pdf]

Supplementary Materials

Structure-Based Design of Potent Peptidomimetic Inhibitors Covalently Targeting SARS-CoV-2 Papain-Like Protease

Qian Wang[1,2,#], Guofeng Chen[2,3,#], Jian He[2,3], Jiameng Li[1,2], Muya Xiong[2,3], Haixia Su[2], Minjun Li[4], Hangchen Hu[5,*], Yechun Xu[1,2,3,5,*]

[1]School of Chinese Materia Medica, Nanjing University of Chinese Medicine, Nanjing 210023, China

[2]State Key Laboratory of Drug Research, Shanghai Institute of Materia Medica, Chinese Academy of Sciences, Shanghai 201203, China

[3]University of Chinese Academy of Sciences, Beijing 100049, China

[4]Shanghai Synchrotron Radiation Facility, Shanghai Advanced Research Institute, Chinese Academy of Sciences, Shanghai 201210, China

[5]School of Pharmaceutical Science and Technology, Hangzhou Institute for Advanced Study, University of Chinese Academy of Sciences, Hangzhou 310024, China

[#]These authors contributed equally

*Correspondence: hangchenhu@ucas.ac.cn; ycxu@simm.ac.cn

Table of contents

Figure S1 The inhibition profile for GRL0617..3

Figure S2 A diagram of workflow and conclusions...3

Figure S3 ^{1}H NMR Spectra for compound **2b**...4

Figure S4 ^{13}C NMR Spectra for compound **2b**..4

Figure S5 HRMS-ESI for compound **2b** 5

Figure S6 ^{1}H NMR Spectra for compound **2d**..5

Figure S7 ^{13}C NMR Spectra for compound **2d**..6

Figure S8 HRMS-ESI for compound **2d** 6

Figure S9 ^{1}H NMR Spectra for compound **2**..7

Figure S10 ^{13}C NMR Spectra for compound **2**..7

Figure S11 HRMS-ESI for compound **2**...8

Figure S12 HPLC traces for compound **2**...8

Figure S13 ^{1}H NMR Spectra for compound **4**...9

Figure S14 ^{13}C NMR Spectra for compound **4**..10

Figure S15 HRMS-ESI for compound **4**...10

Figure S16 HPLC traces for compound **4**...11

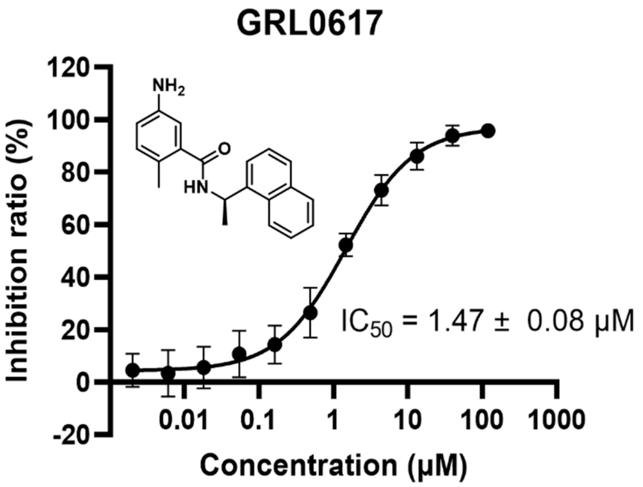

Figure S1. The chemical structure and inhibition profile for GRL0617 against SARS-CoV-2 PL^{pro} with RLRGG-AMC substrate.

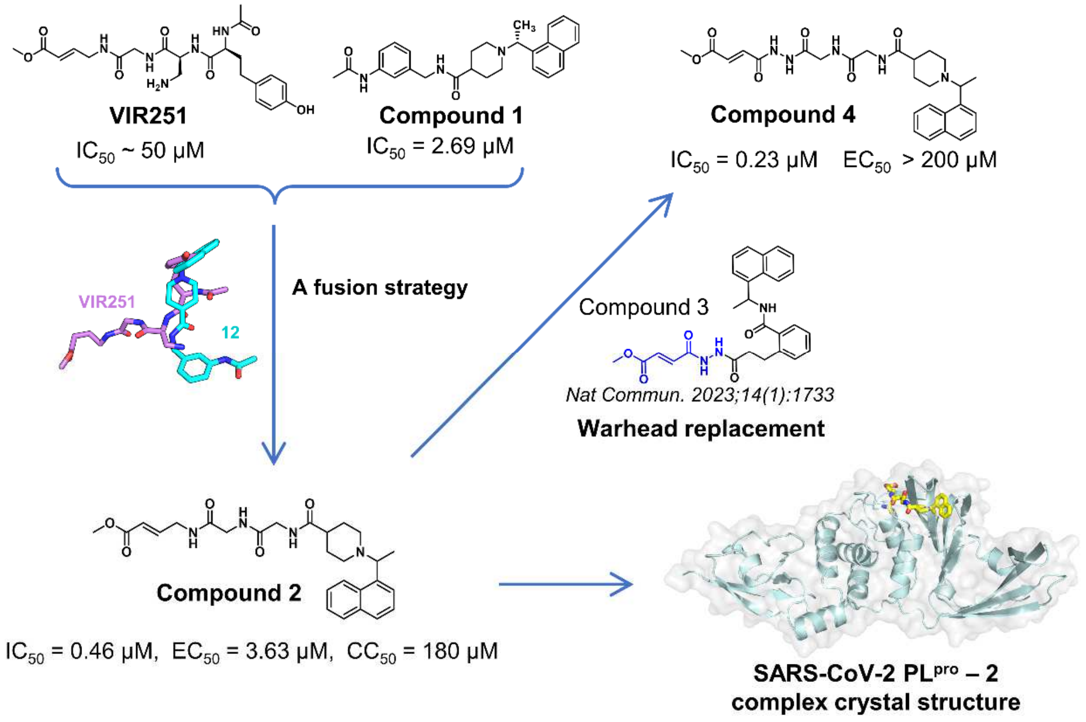

Figure S2. A diagram of workflow and conclusions.

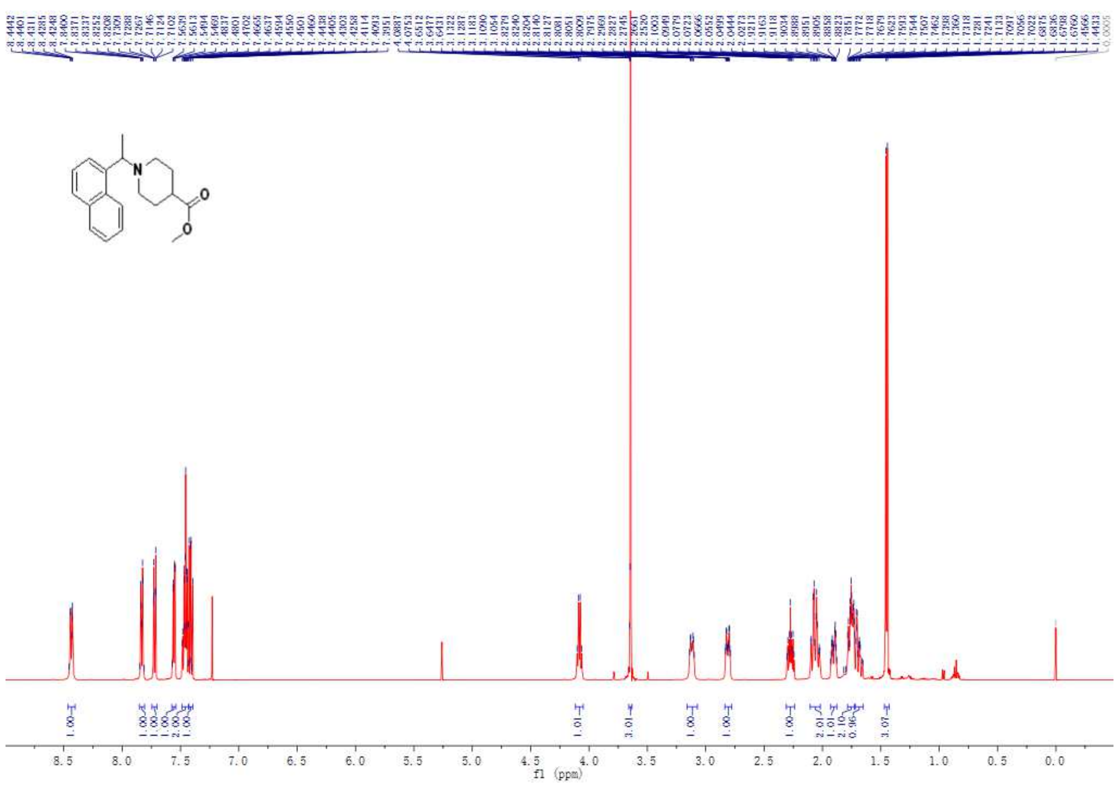

Figure S3. ^{1}H NMR Spectra for compound **2b**.

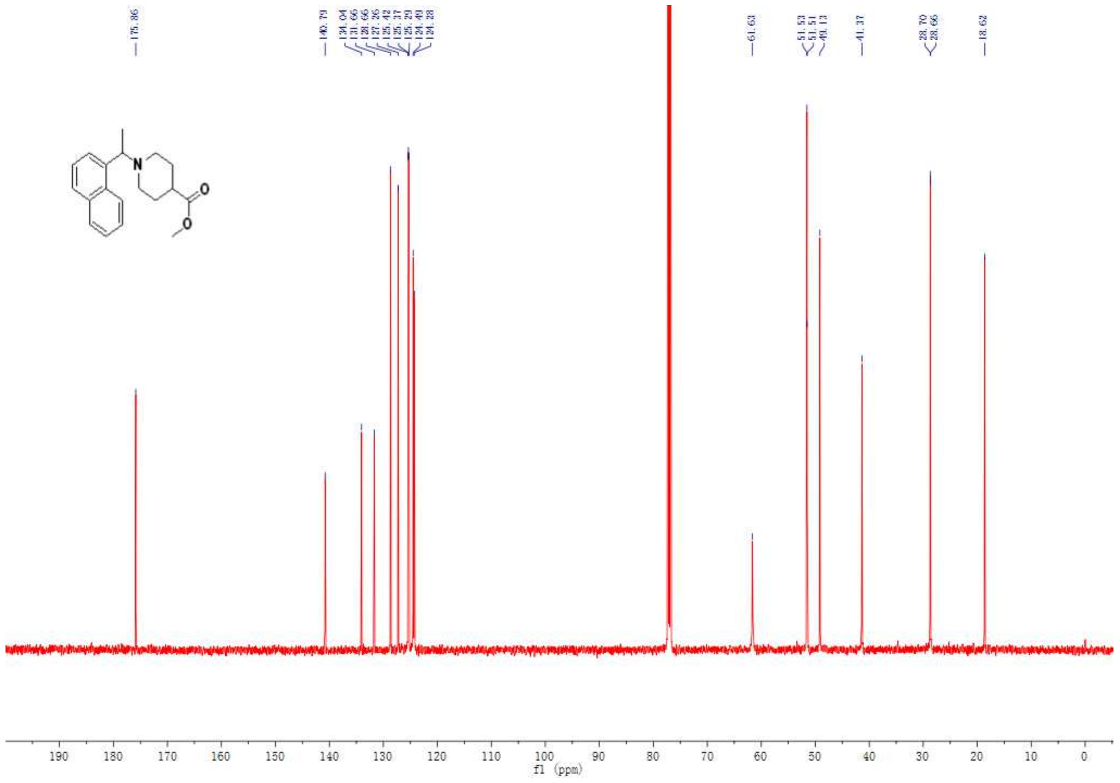

Figure S4. ^{13}C NMR Spectra for compound **2b.**

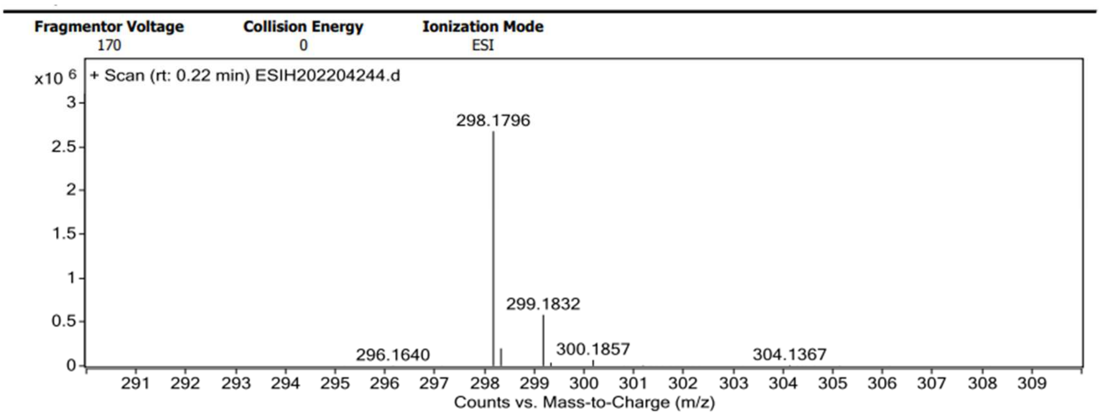

Figure S5. HRMS-ESI for compound **2b.**

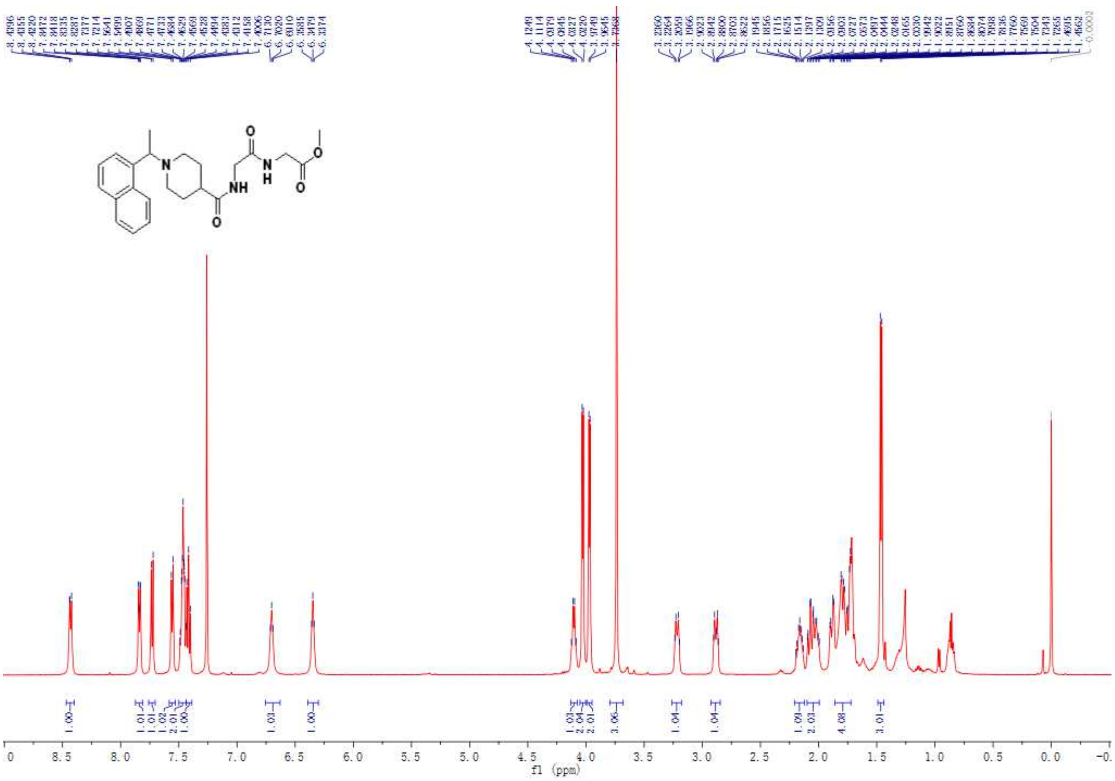

Figure S6. ^{1}H NMR Spectra for compound **2d.**

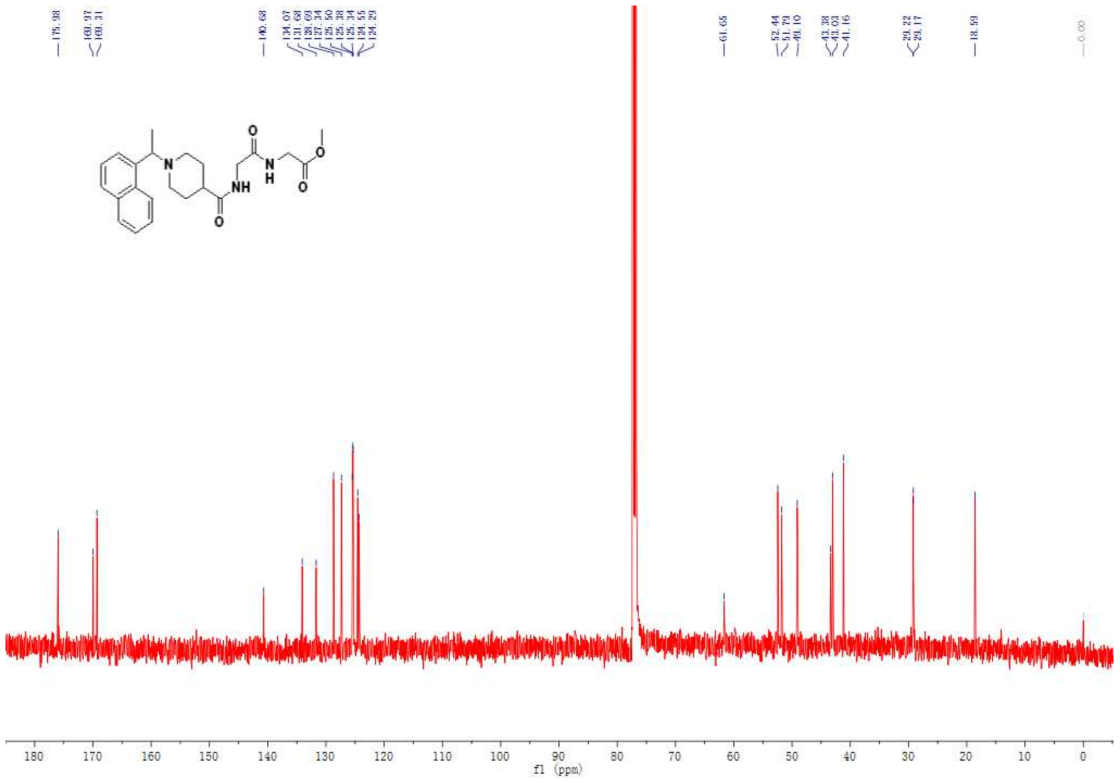

Figure S7. ^{13}C NMR Spectra for compound **2d.**

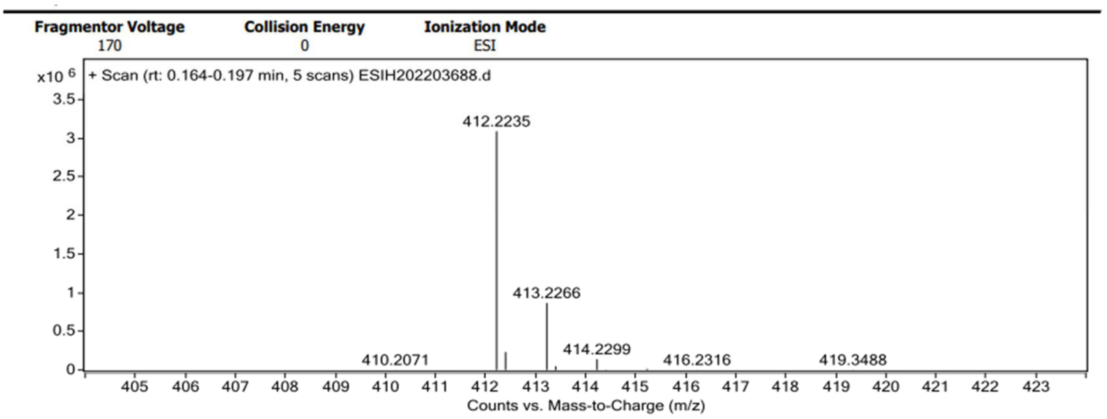

Figure S8. HRMS-ESI for compound **2d.**

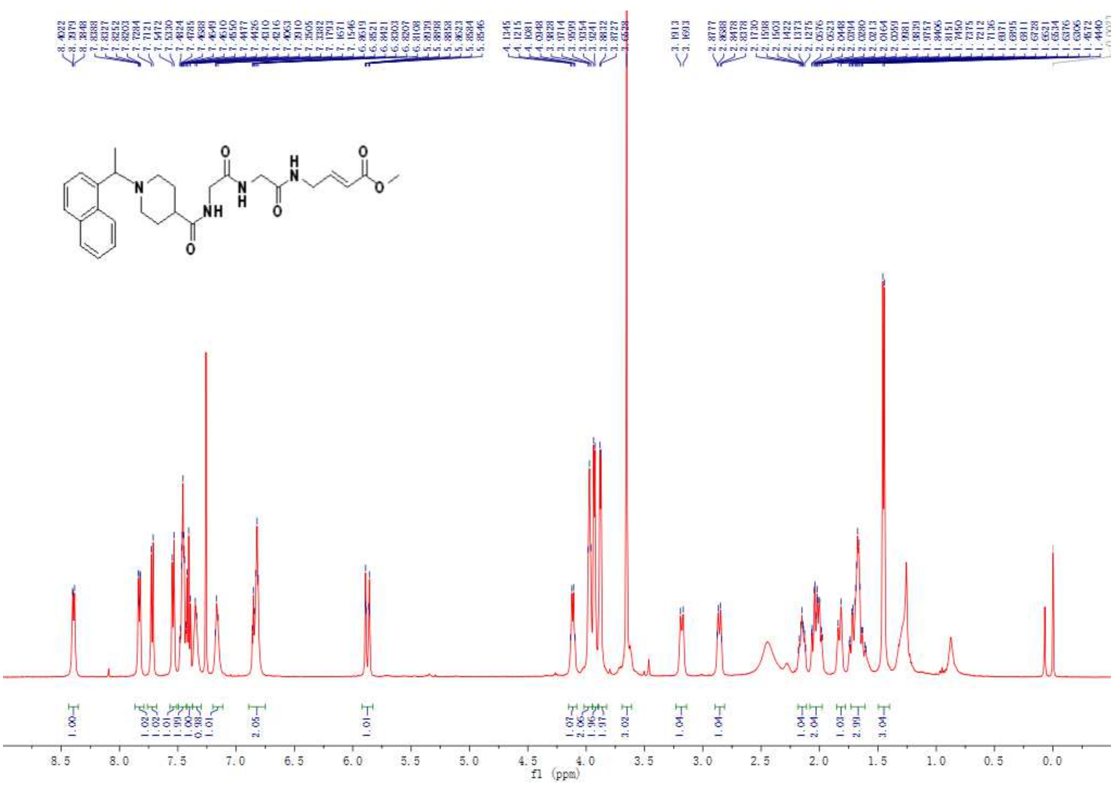

Figure S9. ^{1}H NMR Spectra for compound **2.**

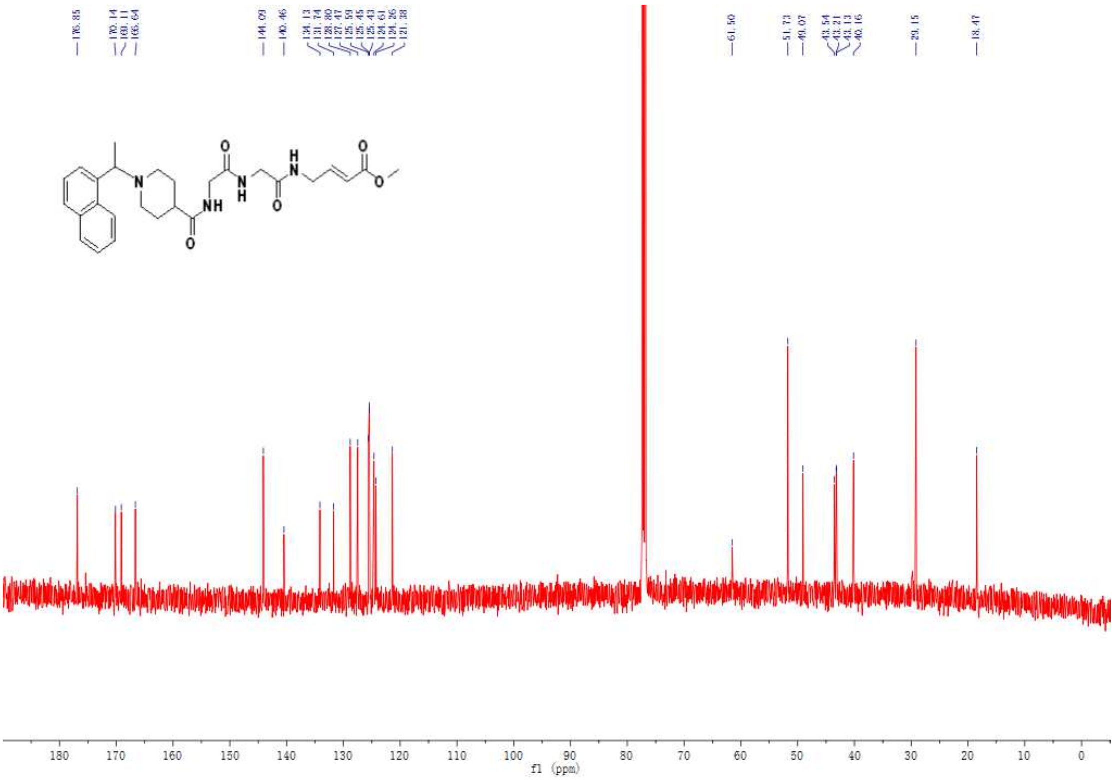

Figure S10. ^{13}C NMR Spectra for compound **2.**

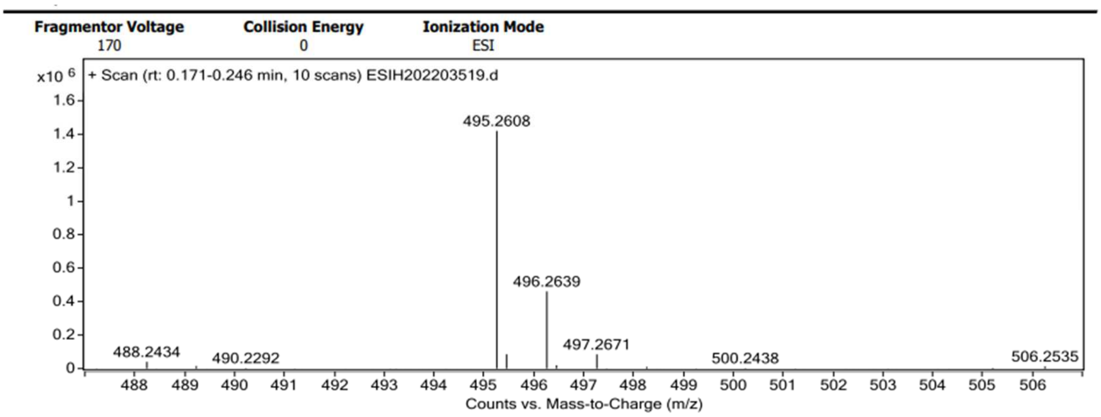

Figure S11. HRMS-ESI for compound **2**

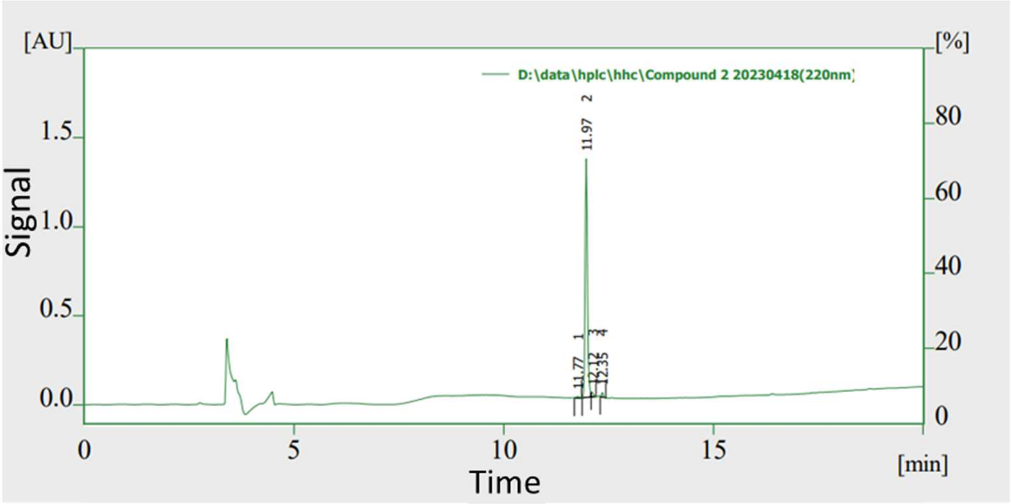

Results Table：

	Retention Time [min]	Peak Area [mAU.s]	Area [%]	Peak Height [mAU]
1	11.767	32.990	0.6	9.545
2	11.967	5101.261	95.7	1339.993
3	12.117	110.727	2.1	22.794
4	12.350	83.274	1.6	25.261
	Total	5328.252	100.0	1397.593

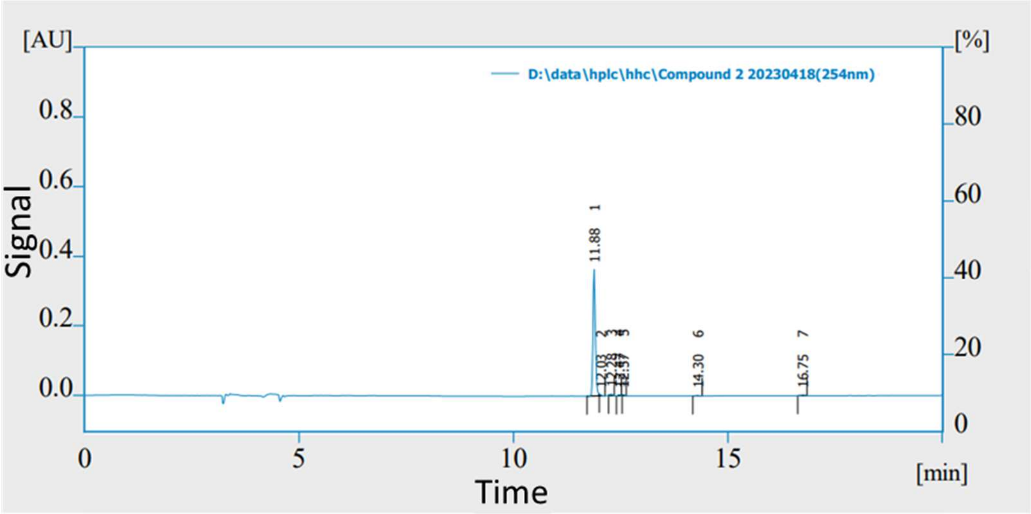

Results Table:

	Retention Time [min]	Peak Area [mAU.s]	Area [%]	Peak Height [mAU]
1	11.883	1417.414	96.0	364.688
2	12.033	15.761	1.1	3.068
3	12.283	14.680	1.0	4.216
4	12.467	8.598	0.6	2.792
5	12.567	7.218	0.5	2.303
6	14.300	5.333	0.4	1.188
7	16.750	8.094	0.5	1.656
	Total	1477.098	100.0	379.910

Figure S12. HPLC traces for compound **2**

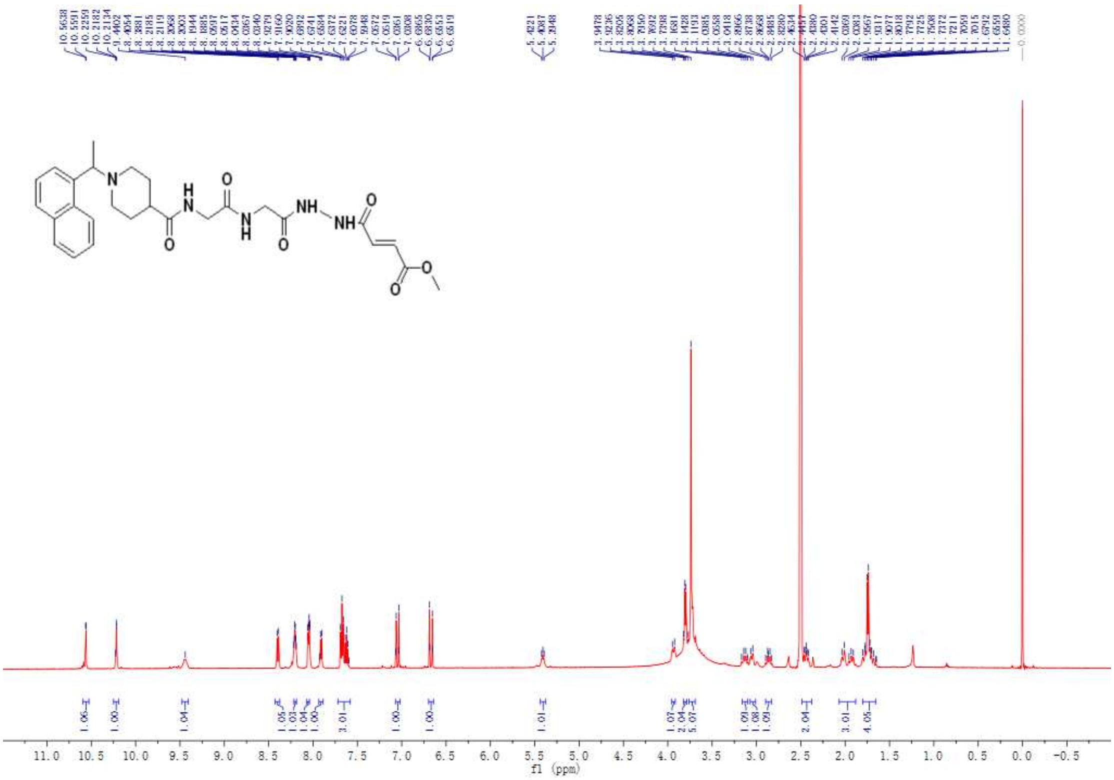

Figure S13. ^{1}H NMR Spectra for compound **4.**

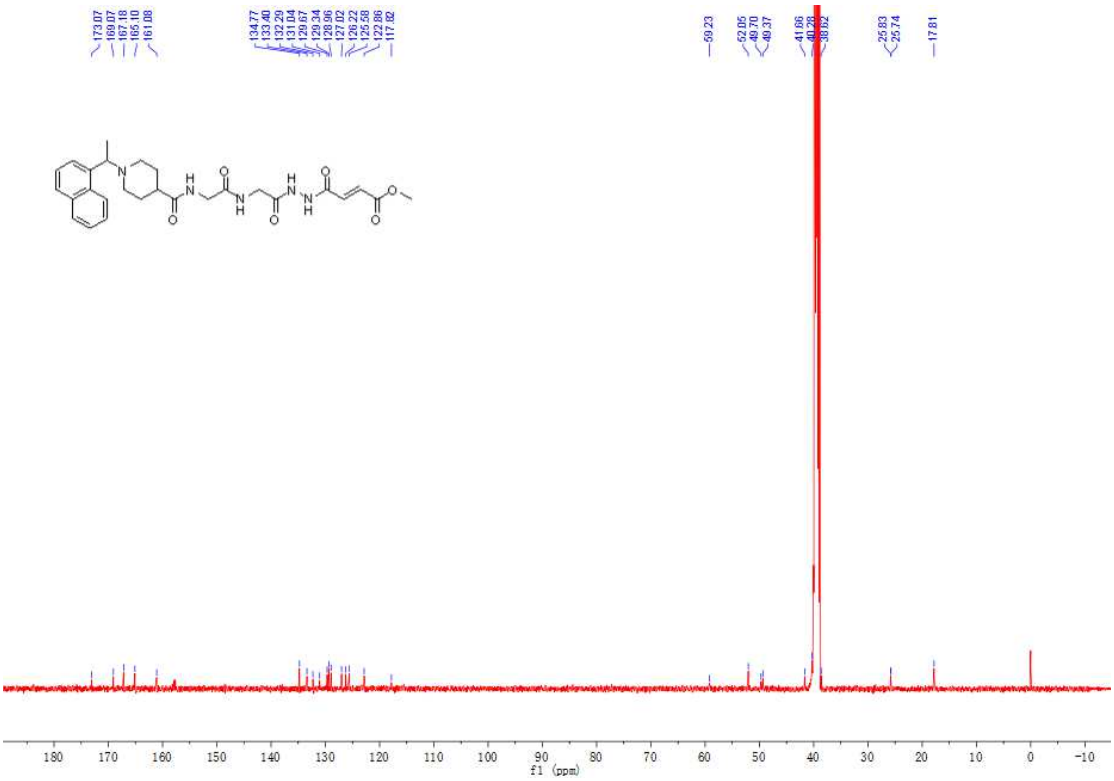

Figure S14. ^{13}C NMR Spectra for compound **4.**

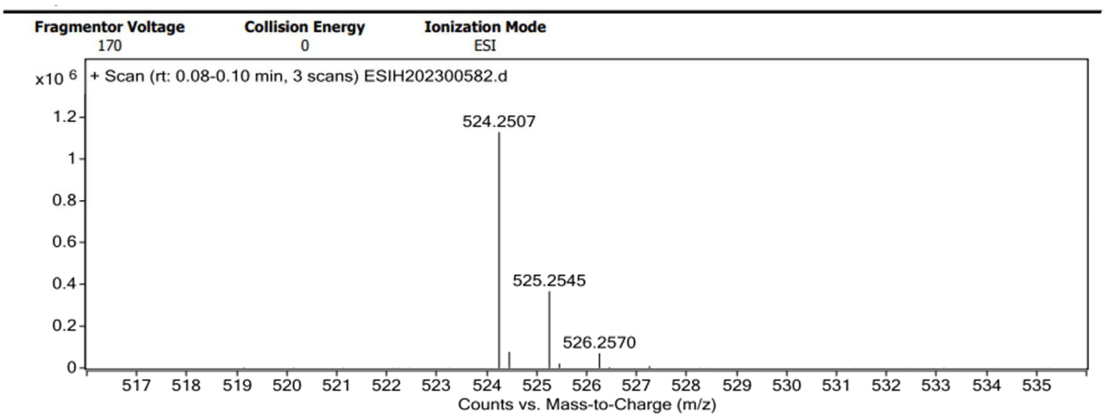

Figure S15. HRMS-ESI for compound **4.**

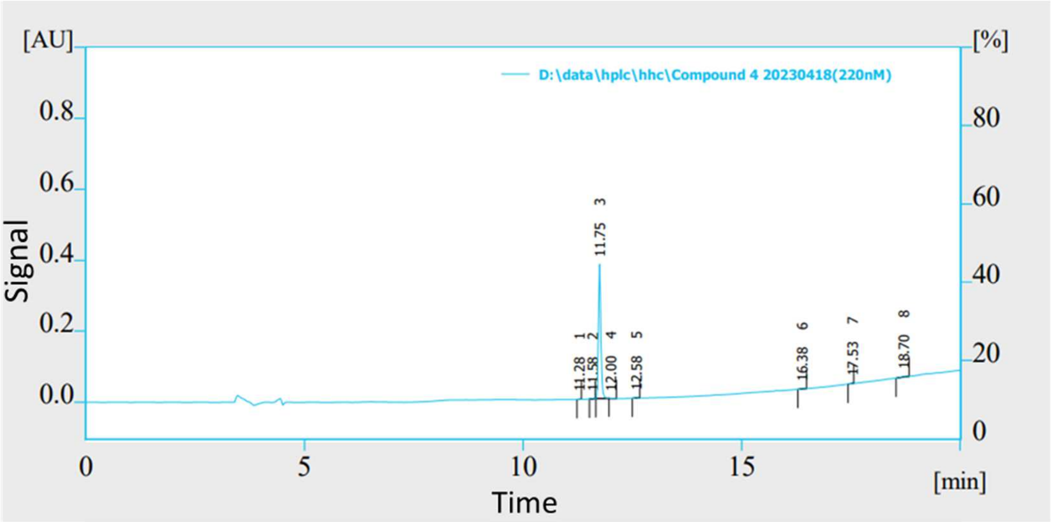

Results Table

	Retention Time [min]	Peak Area [mAU.s]	Area [%]	Peak Height [mAU]
1	11.283	2.238	0.1	0.774
2	11.583	6.362	0.4	1.836
3	11.750	1469.927	97.0	379.153
4	12.000	9.065	0.6	1.373
5	12.583	8.370	0.6	2.110
6	16.383	4.572	0.3	1.188
7	17.533	1.472	0.1	0.327
8	18.700	13.916	0.9	2.267
	Total	1515.922	100.0	389.029

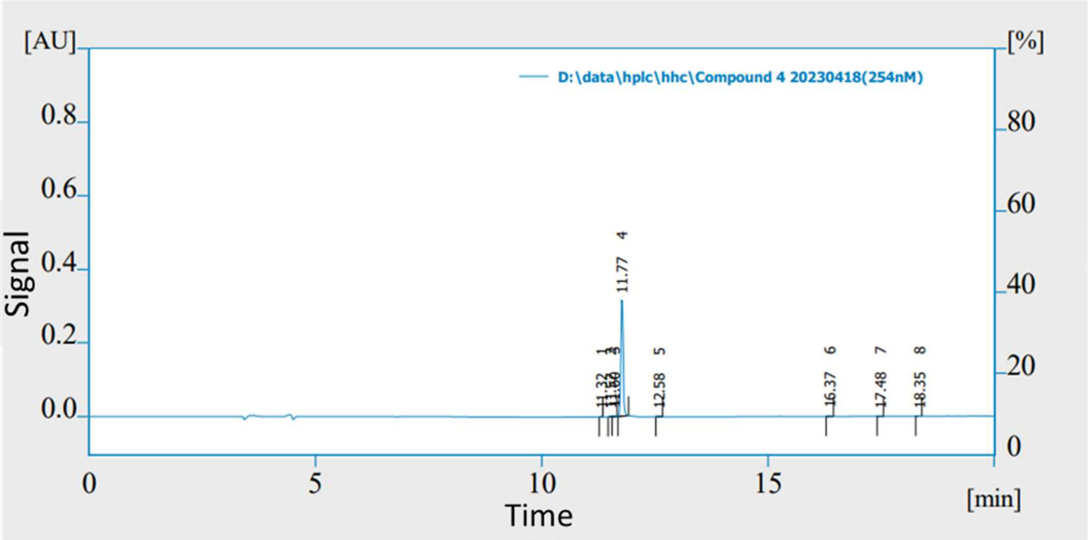

Results Table：

	Retention Time [min]	Peak Area [mAU.s]	Area [%]	Peak Height [mAU]
1	11.317	1.568	0.1	0.619
2	11.517	4.117	0.3	1.471
3	11.600	12.635	1.1	3.119
4	11.767	1158.280	96.8	314.900
5	12.583	3.176	0.3	0.803
6	16.367	9.186	0.8	2.185
7	17.483	4.656	0.4	1.221
8	18.350	3.324	0.3	0.952
	Total	1196.942	100.0	325.270

Figure S16. HPLC traces for compound **4**
